# Feasibility and safety of weaning premature infants from nasal continuous positive airway pressure to high-flow nasal cannula: a prospective observational case study

**DOI:** 10.1186/s12887-024-05167-2

**Published:** 2024-11-15

**Authors:** Shu-Ting Yang, Hao-Wei Chung, Hsiu-Lin Chen

**Affiliations:** 1 Division of Neonatology, Department of Pediatrics, Kaohsiung Medical University Hospital, Kaohsiung Medical University, No. 100, Tzyou 1st Road Kaohsiung 807, Kaohsiung, Taiwan; 2 Department of Respiratory Therapy, College of Medicine, Kaohsiung Medical University, No. 100, Shiquan 1st Road Kaohsiung 807, Kaohsiung, Taiwan

**Keywords:** Nasal continuous positive airway pressure, High-flow nasal cannula, Premature infant

## Abstract

**Background:**

Nasal continuous positive airway pressure (NCPAP) is widely used for premature infants with respiratory distress syndrome (RDS). A high-flow nasal cannula (HFNC) provides positive end-expiratory pressure using high-flow oxygen; however, the variability in distending pressure is a primary concern. This study evaluated the feasibility and safety of a newly designed protocol for NCPAP weaning with cyclic HFNC use for premature infants.

**Methods:**

Premature infants with RDS using NCPAP support who were ready for weaning were enrolled. The weaning protocol used cyclic NCPAP with HFNC every 3 h for 3 days in the neonatal intensive care unit. The heart rate (HR), respiratory rate (RR), pulse oximetry (SpO_2_), transcutaneous carbon dioxide (PtcCO_2_), and cerebral tissue oxygen saturation (StO_2_) at the end of NCPAP with HFNC support were recorded once daily for 3 days.

**Results:**

From June 2019 to April 2021, 46 premature infants (27 male, 19 female) were enrolled. The mean gestational age and birth body weight were 28.7 ± 2.6 weeks and 1181 ± 354 g, respectively. No statistically significant differences in the HR, RR, SpO_2_, and cerebral StO_2_ during NCPAP weaning with HFNC were observed. However, the mean PtcCO_2_ with NCPAP was statistically significantly lower than that with HFNC (46.9 ± 6.0 mmHg vs. 47.9 ± 6.4 mmHg, *P* = 0.02).

**Conclusions:**

The feasibility and safety of the NCPAP weaning protocol with cyclic HFNC for premature infants are acceptable in this preliminary study. Due to the limited number of participants, further studies are required for more comprehensive analysis.

**Trial registration:**

This prospective observational case study was approved by the Human Experiment and Ethics Committee of our hospital (approval number: KMUHIRB-SV(I)-20180059; approval date: January 11, 2019).

## Background

Respiratory distress syndrome (RDS) is a common cause of neonatal intensive care unit (NICU) admission for premature infants [1–3]. Non-invasive respiratory support for premature infants includes nasal intermittent positive pressure ventilation, nasal continuous positive airway pressure (NCPAP), and high-flow nasal cannula (HFNC) use. NCPAP has been widely used to treat RDS in premature infants by providing continuous positive pressure to prevent alveolar prolapse and stabilize functional residual capacity [1–3]. However, the need for prongs that completely fit the nostrils may damage the nasal mucosa and septum, and the higher positive airway pressure may also induce complications such as pneumothorax, pneumomediastinum, and abdominal distension [1, 2].

Conversely, the HFNC provides positive end-expiratory pressure (PEEP) using high-flow oxygen (2–8 L/min) for infants [1, 2, 4, 5]. The advantages of the HFNC include a lower risk of injury to the nares, increased comfort, and ease of use. However, the variability in PEEP is one of the primary concerns associated with HFNC use [1, 2, 4, 5]. The HFNC has been considered an NCPAP weaning device (step-down) for premature infants [1, 2, 4, 5]. This study aimed to design and evaluate the feasibility and safety of a new protocol involving cyclic HFNC use for NCPAP weaning among premature infants.

## Methods

### Study population

We designed a prospective observational study involving premature infants (gestational age [GA] < 37 weeks) with RDS using NCPAP support who were ready for weaning. Participants were enrolled between June 6, 2019 and April 13, 2021. The evaluation criteria for NCPAP weaning included a fraction of inspired oxygen (FiO_2_) of 0.21, PEEP of 4–5 cm H_2_O, with relatively stable vital signs, and no episodes of apnea while supported by NCPAP support for 3 days. The exclusion criteria were term infants and preterm infants with major birth defect. The major birth defect included chromosome anomalies, congenital heart defects, neural tube defects, and congenital gastrointestinal defects (such as gastroschisis or omphalocele).

### Ethics

This prospective observational case study was approved by the Human Experiment and Ethics Committee of our hospital (approval number: KMUHIRB-SV(I)-20180059; approval date: January 11, 2019). All experiments were performed in accordance with relevant guidelines and regulations. Written informed consent was obtained from the parents of the included premature infants.

### Study design

**Fig. 1:**
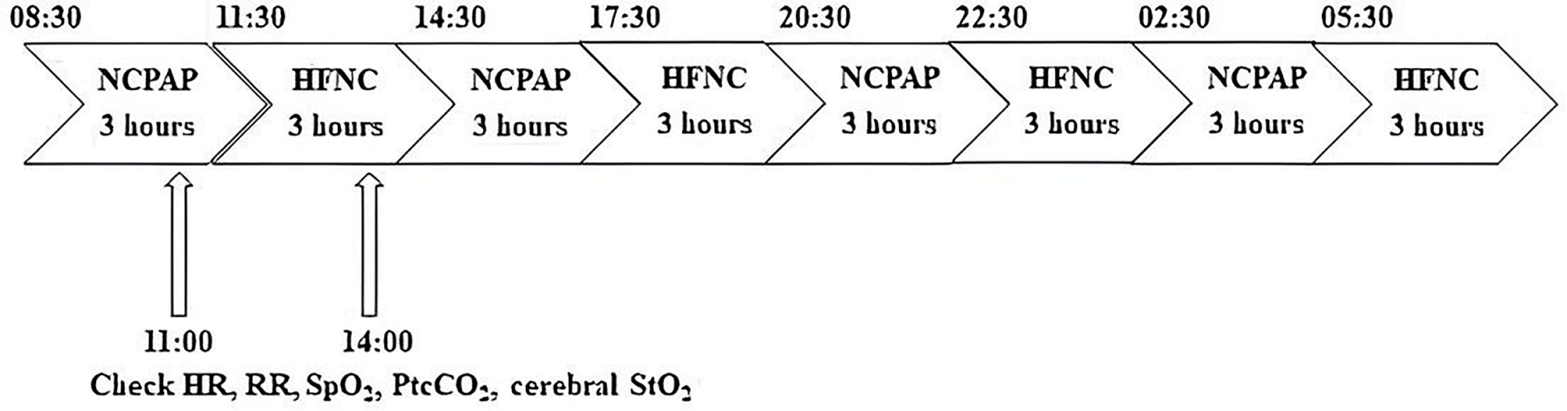
Daily weaning protocol for 3 days

The 3-day weaning protocol comprised alternating 3-hour sessions of NCPAP and cyclic HFNC in each participant in the NICU at our hospital, beginning from 08:30 AM with NCPAP. The flow rate of HFNC was adjusted to match the pressure levels during its use, as measured by a GiO Digital Pressure Gauge (GIO 6, GaleMed Corporation, Taipei, Taiwan) at the distal end of the nasal cannula. The pressure was set to correspond with the PEEP used during the preceding NCPAP treatment. The flow rate of HFNC was maintained at 4–6 L per minute throughout the study. The heart rate (HR), respiratory rate (RR), pulse oximetry (SpO_2_), transcutaneous carbon dioxide (PtcCO_2_), and cerebral tissue oxygen saturation (StO_2_) were recorded for 0.5 h. Then, the respiratory support was changed to HFNC use for 3 h starting at 11:30 AM. We again recorded the HR, RR, SpO_2_, PtcCO_2_, and cerebral StO_2_ for 0.5 h (Fig. 1). The primary outcomes were the differences in the mean values of HR, RR, SpO_2_, PtcCO_2_, and cerebral StO_2_ recorded during NCPAP and HFNC. After cyclic use for 3 days, neonatologists in the NICU evaluated the clinical condition of the infants and assessed if they could tolerate continuous HFNC support until complete weaning was achieved.

This cyclic approach was chosen in line with the standard nursing care schedule in our NICU, which follows a 3-hour cycle. We also believed that providing respiratory support via HFNC in 3-hour intervals would offer more consistent support to preterm infants than the shorter cycles would [6]. The initiation of cyclic HFNC use for 3 days in enrolled preterm infants was based on the inclusion criterion of no apnea occurrences while on NCPAP support for 3 consecutive days. We monitored the frequency of apnea during the cyclic use of NCPAP and HFNC. The absence of apnea during the 3-day cyclic period was considered indicative of successful weaning from NCPAP to HFNC.

However, some infants could not successfully wean from NCPAP using the HFNC during the protocol due to observed respiratory distress, which included a heart rate (HR) > 160 beats per min, a respiratory rate (RR) over 60 cycles per min, an oxygen saturation (SpO_2_) below 90%, or PtcCO_2_ above 60 mmHg during the 3-day weaning period. Therefore, we further divided the study participants into those successfully weaned from NCPAP using the HFNC within 3 days (success group) and those weaned after > 3 days (failure group).

Data on the sex, GA, birth body weight (BW), delivery mode, Neonatal Therapeutic Intervention Scoring System score, Apgar score, medication used to treat apnea, respiratory therapy condition, post-menstrual age (PMA), BW when starting weaning from NCPAP, apnea frequency, vital sign changes, and possible adverse effects associated with the HFNC of all enrolled infants were collected.

### Study devices

This study used the Optiflow System HFNC (Fisher & Paykel Optiflow System Healthcare, Auckland, New Zealand); short binasal prongs with different sizes were chosen based on the infant’s BW. NCPAP was administered using the Babi.Plus^®^ Bubble CPAP system (GaleMed Corporation, Taipei, Taiwan). The SenTec Digital Monitoring System (SenTec AG, Therwil, Switzerland) was used to measure PtcCO_2_. The FORE-SIGHT Oximeter MC-2000 Series Cerebral Oximeter (CAS Medical Systems, Inc., Branford, CT, USA) was used to measure cerebral StO_2_.

### Statistics

Data recording and evaluation were performed using JMP 10 software (SAS Institute Inc., Cary, NC, USA). The rank sum test was performed to compare numerical variables of primary outcomes during NCPAP and HFNC and the characteristics and clinical outcomes in the success and failure groups. Univariate regression analyses were performed to analyze the factors associated with the PtcCO_2_ of preterm infants using NCPAP and HFNC. The chi-square test was performed to compare categorical variables in the success and failure groups. In contrast, the rank sum test was performed to compare numerical variables of primary outcomes, respiratory outcomes, and prognoses in the success and failure groups.

## Results

**Fig. 2:**
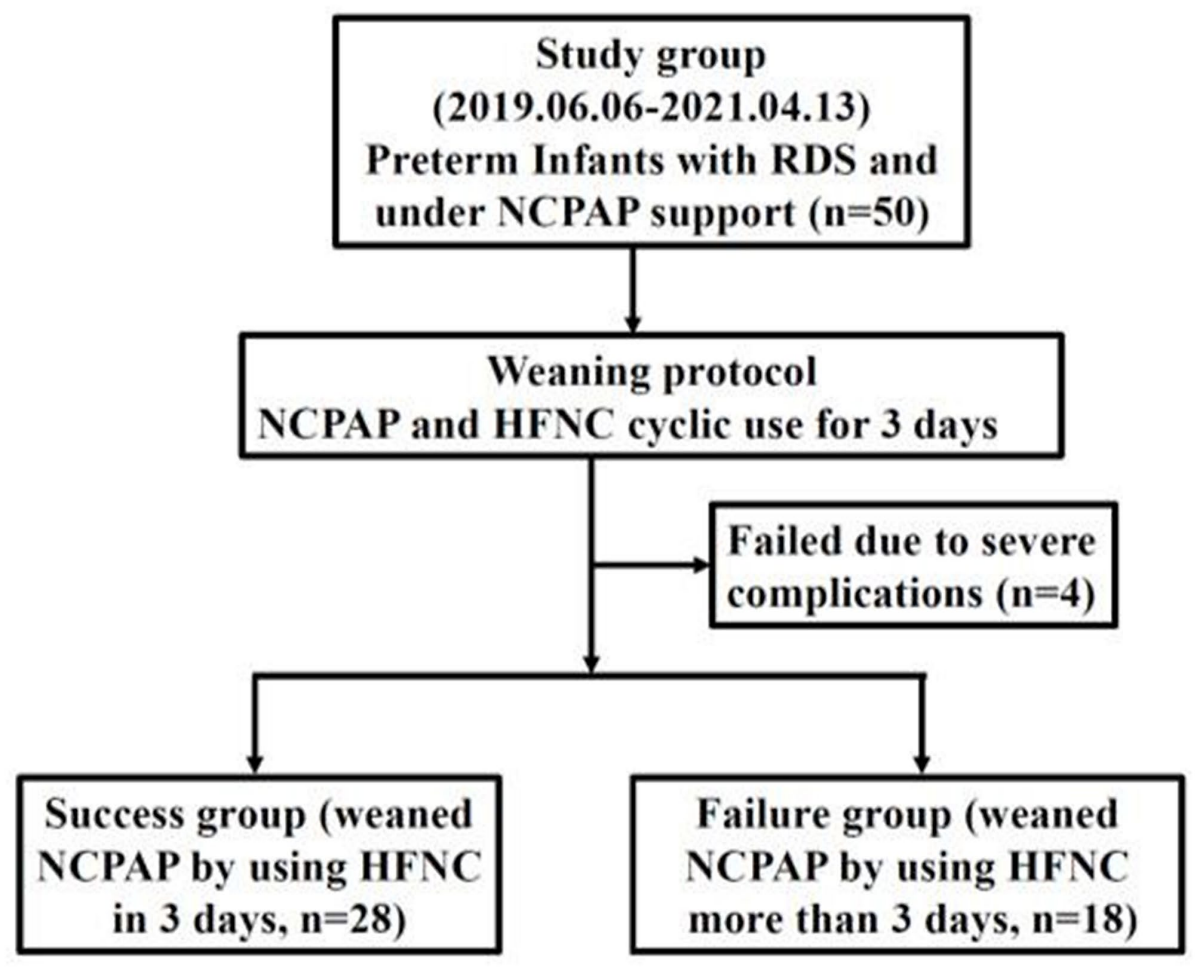
Flow chart of the study design

Fifty premature infants admitted to the NICU at our university hospital who met the inclusion criteria underwent the weaning protocol after written informed consent was received from their parents. Four participants were excluded due to severe complications, such as severe pulmonary hypertension or hydrocephalus (Fig. 2). Therefore, 46 participants were included in this study. No adverse effects were observed among the remaining 46 infants. The mean GA was 28.7 weeks (standard deviation (SD), ± 2.6 weeks), whereas the mean birth BW was 1181 g (SD, ± 354 g). The characteristics and clinical outcomes of the 46 infants are shown in Table 1.

**Table 1:** Characteristics and clinical outcomes of infants

Item	*N*	%
Premature	46	100
GA (weeks)		
< 28	18	39
28 to < 32	22	48
Birth BW (g)		
≤ 1000	16	35
1001–1499	20	43
PMA (weeks) when involved		
< 37	13	28
Male	27	59
VD	29	6
NTISS score ≥ 15	42	91
5-min Apgar score ≤ 7	18	39
Respiratory distress syndrome		
Grade 2	11	24
Grade 3	26	57
Grade 4	9	19
Surfactant usage	23	50
Intubation after birth	20	43
Medicine for apnea		
Theophylline	20	43
Caffeine	16	35
Packed RBC transfusion	36	78
Moderate-to-severe BPD	20	43
PDA	30	65
Without treatment	6	13
With conservative treatment	24	52
Late-onset sepsis	16	35
Blood culture proved	9	20
Clinical diagnosis	7	15
Hypotension	12	26
IVH grade ≥ III	3	7
ROP stage ≥ III	7	15
NEC stage ≥ II	1	2

GA, gestational age; BW, birth body weight; PMA, post-menstrual age; VD, vaginal delivery; NTISS, Neonatal Therapeutic Intervention Scoring System; RBC, red blood cell; BPD, bronchopulmonary dysplasia; PDA, patent ductus arteriosus; IVH, intraventricular hemorrhage; ROP, retinopathy of prematurity; NEC, necrotizing enterocolitis

The mean PMA during NCPAP weaning was 35.6 weeks (SD, ± 2.3 weeks), and the mean BW was 1990 g (SD, ± 527 g). The mean apnea frequency during the 3-day cyclic use of NCPAP and HFNC was less than once daily. The primary outcomes (mean ± SD) of 46 enrolled infants, in whom NCPAP and HFNC support were administered, revealed no statistical differences in the HR (162 ± 13 beats per min under NCPAP, 163 ± 13 beats per min under HFNC, *P* = 0.34), RR (46 ± 12 cycles per min under NCPAP, 46 ± 13 cycles per min under HFNC, *P* = 0.71), SpO_2_ (97 ± 3% under NCPAP, 97 ± 3% under HFNC, *P* = 0.46), and cerebral StO_2_ (75.3 ± 5.8% under NCPAP, 74.9 ± 5.2% under HFNC, *P* = 0.58) during NCPAP weaning with HFNC. The median of SpO_2_ and cerebral StO_2_ under NCPAP and HFNC were 97% vs. 97% and 75.8% vs. 75.5%. The PtcCO_2_ was higher in this population with HFNC support (47.9 ± 6.4 mmHg) than with NCPAP support (46.9 ± 6.0 mmHg; *P* = 0.02).

Table 2 shows the associated factors influencing the differences in PtcCO_2_ with cyclic use of NCPAP and HFNC on univariate regression analysis. None of these factors affected the differences in PtcCO_2_.

**Table 2:** Factors influencing differences in PtcCO_2_ with the use of NCPAP and HFNC support

	Differences in PtcCO_2_ (HFNC and NCPAP)			
Item	Regression coefficient	Lower 95% CI	Upper 95% CI	*P*-value
GA	-0.104	-0.424	0.215	0.52
BBW	-0.001	-0.003	0.002	0.52
Female-male*	1.083	-0.556	2.722	0.19
CS-VD†	-0.634	-2.313	1.044	0.46
NTISS	-0.002	-0.208	0.204	0.98
1-min Apgar score	0.032	-0.358	0.422	0.87
5-min Apgar score	0.023	-0.329	0.375	0.89
RDS grade	0.918	-0.306	2.142	0.14
Surfactant usage	-1.017	-2.632	0.597	0.22
Intubation after birth	-0.624	-2.424	1.176	0.49
Apnea medication				
Used-not used‡	-1.582	-3.533	0.368	0.11
Caffeine-theophylline§	-0.508	-2.296	1.280	0.58
Packed RBC transfusion	1.054	-0.906	3.014	0.29
Moderate-to-severe BPD	0.289	-1.348	1.926	0.73
PDA				
Without Tx	-0.933	-3.339	1.472	0.44
With conservative Tx	-0.001	-1.626	1.624	0.99
Late-onset sepsis	0.886	-0.812	2.584	0.30
Blood culture-proven	0.147	-1.899	2.193	0.89
Clinical diagnosis	1.548	-0.697	3.793	0.18
Hypotension	0.202	-1.646	2.051	0.83
IVH grade ≥ III	-0.226	-3.515	3.062	0.89
ROP stage ≥ III	0.728	-1.529	2.985	0.53
NEC stage ≥ II	0.601	-4.965	6.168	0.83

*Female infants compared with male ones

†CS compared with VD

‡Used compared with not used

§Caffeine compared with aminophylline

NCPAP, nasal continuous positive airway pressure; HFNC, high-flow nasal cannula; PtcCO_2_, transcutaneous carbon dioxide; CI, confidence interval; GA, gestational age; BBW, birth body weight; CS, cesarean section; VD, vaginal delivery; NTISS, Neonatal Therapeutic Intervention Scoring System; RDS, respiratory distress syndrome; RBC, red blood cell; BPD, bronchopulmonary dysplasia; PDA, patent ductus arteriosus; Tx, treatment; IVH, intraventricular hemorrhage; ROP, retinopathy of prematurity; NEC, necrotizing enterocolitis

Eighteen premature infants could not be completely weaned from NCPAP to HFNC after 3 days of protocol and required an extended period of weaning (defined as the failure group). There were no statistical differences in infants’ characteristics and clinical outcomes in the success and failure groups (Table 3). The mean apnea frequency during the 3-day course of cyclic use of NCPAP and HFNC in both groups was less than once daily.

**Table 3:** Characteristics and clinical outcomes of enrolled infants in the success and failure groups

Item	*Success group (*n* = 28)	†Failure group (*n* = 18)	*P*-value
GA (weeks) (mean ± SD)	29.2 ± 2.4	27.9 ± 2.6	0.16
Birth BW (g) (mean ± SD)	1201 ± 317	1150 ± 412	0.44
PMA at the beginning of weaning (weeks) (mean ± SD)	35.3 ± 2.4	35.9 ± 2.2	0.33
Male, n (%)	17 (61)	10 (56)	0.73
Vaginal delivery, n (%)	17 (61)	13 (72)	0.68
NTISS (mean ± SD)	18 ± 4	20 ± 4	0.14
1-min Apgar score (mean ± SD)	4 ± 2	4 ± 2	0.91
5-min Apgar score (mean ± SD)	6 ± 2	6 ± 2	0.74
RDS grade (mean ± SD)	3 ± 0	3 ± 1	0.35
Surfactant usage	13 (46)	10 (56)	0.55
Intubation after birth	12 (43)	8 (44)	0.92
Medicine for apnea, n (%)			
Theophylline	11 (39)	9 (50)	0.47
Caffeine	11 (39)	5 (28)	0.42
Packed RBC transfusion	21 (58)	15 (42)	0.50
Moderate-to-severe BPD, n (%)	10 (36)	10 (56)	0.19
PDA, n (%)	17 (61)	13 (72)	0.42
Without Tx	3 (11)	3 (17)	0.56
With conservative Tx	14 (50)	10 (55)	0.71
Late-onset sepsis, n (%)	7 (25)	9 (50)	0.08
Blood culture proved	3 (11)	6 (33)	0.059
Clinical diagnosis	4 (14)	3 (17)	0.83
Hypotension, n (%)	7 (25)	5 (28)	0.83
IVH grade ≥ III, n (%)	2 (7)	1 (6)	0.83
ROP stage ≥ III, n (%)	5 (18)	2 (11)	0.53
NEC stage ≥ II, n (%)	1 (4)	0 (0)	0.42

*Success group: infants weaned from NCPAP successfully using the HFNC for 3 days

†Failure group: infants weaned from NCPAP using the HFNC for > 3 days

GA, gestational age; SD, standard deviation; BW, body weight; PMA, post-menstrual age; NTISS, Neonatal Therapeutic Intervention Scoring System; RDS, respiratory distress syndrome; RBC, red blood cell; BPD, bronchopulmonary dysplasia; PDA, patent ductus arteriosus; Tx, treatment; IVH, intraventricular hemorrhage; ROP, retinopathy of prematurity; NEC, necrotizing enterocolitis

Table 4 presents the primary outcomes of infants in the success and failure groups with NCPAP or HFNC support. The median of SpO_2_ under NCPAP or HFNC in the success and failure groups were 98% vs. 97% and 98% vs. 97%, respectively. The median of cerebral StO_2_ under NCPAP or HFNC in the success and failure groups were 76.0% vs. 75.7% and 75.9% vs. 74.6%, respectively. The cerebral StO_2_ with HFNC support was lower for infants in the failure group than in the other groups.

**Table 4:** Primary outcomes of enrolled infants in the success and failure groups

Respiratory support	Item	Mean ± SD		P-value
		*Success group (*n* = 28)	†Failure group (*n* = 18)	
NCPAP	HR (bpm)	165 ± 12	158 ± 13	< 0.001*
	RR (cpm)	46 ± 13	45 ± 11	0.75
	SpO_2_ (%)	97 ± 2	97 ± 3	0.84
	PtcCO_2_ (mmHg)	46.6 ± 6.0	47.4 ± 6.1	0.89
	Cerebral StO_2_ (%)	75.8 ± 5.2	75.2 ± 5.7	0.40
HFNC	HR (bpm)	166 ± 12	159 ± 14	0.008*
	RR (cpm)	44 ± 12	47 ± 13	0.25
	SpO_2_ (%)	97 ± 2	96 ± 3	0.13
	PtcCO_2_ (mmHg)	47.2 ± 6.3	49.1 ± 6.3	0.09
	Cerebral StO_2_ (%)	75.7 ± 5.6	73.9 ± 4.4	0.023*

*Success group: infants weaned from NCPAP successfully using the HFNC for 3 days

†Failure group: infants weaned from NCPAP using the HFNC for > 3 days

NCPAP, nasal continuous positive airway pressure; HFNC, high-flow nasal cannula; SD, standard deviation; HR, heart rate; bpm, beats per minute; RR, respiratory rate; cpm, cycles per minute; SpO_2_, pulse oximetry; PtcCO_2_, transcutaneous carbon dioxide; StO_2_, tissue oxygen saturation

Table 5 shows infants’ respiratory outcomes and prognoses in the success and failure groups. The failure group required more NCPAP weaning days, HFNC usage days, and total days of respiratory therapy. The PMA at discontinuation of NCPAP and HFNC was greater in the failure group than in the success group.

**Table 5:** Respiratory outcomes and prognoses of enrolled infants in the success and failure groups

	Mean ± SD		
Item	*Success group (*n* = 28)	†Failure group (*n* = 18)	*P*-value
PMA at the beginning of weaning (weeks)	35.3 ± 2.4	35.9 ± 2.2	0.33
BW at the beginning of weaning (g)	1907 ± 500	2121 ± 564	0.13
NCPAP weaning days‡	3 ± 0	13 ± 14	< 0.001^*^
Total days of NCPAP usage	30 ± 16	39 ± 18	0.09
PMA at discontinuation of NCPAP (weeks)	35.7 ± 2.4	37.9 ± 3.3	0.04^*^
HFNC usage days§	24 ± 16	34 ± 22	0.03^*^
PMA at discontinuation of HFNC (weeks)	38.8 ± 3.8	41.6 ± 4.7	0.03^*^
Length of hospital stay (days)	88 ± 30	110 ± 46	0.07
PMA at discharge (weeks)	41.7 ± 3.5	43.5 ± 4.8	0.15
Total days of respiratory therapy^‖^	69 ± 28	96 ± 44	0.03^*^

*Success group: infants weaned from NCPAP successfully using the HFNC for 3 days

†Failure group: infants weaned from NCPAP using the HFNC for > 3 days

‡Duration between the start and end of weaning from NCPAP (d)

§Total days of HFNC usage after weaning from NCPAP (d)

‖Total days of respiratory therapy support (including ventilator, nasal intermittent positive pressure ventilation, NCPAP, and HFNC) during admission

SD, standard deviation; PMA, post-menstrual age; BW, body weight; NCPAP, nasal continuous positive airway pressure; HFNC, high-flow nasal cannula

## Discussion

Our study investigated the feasibility and safety of the NCPAP weaning protocol with the cyclic use of an HFNC. This protocol showed no differences in HR, RR, SpO_2_, and cerebral StO_2_; however, the PtcCO_2_ was higher during HFNC use than during NCPAP use. No adverse effects were observed during this study.

The differences in PtcCO_2_ among premature infants during NCPAP and HFNC use in our study may be associated with lower respiratory tract support pressure stability with the HFNC than with NCPAP [4, 5]. Although the difference in PtcCO_2_ levels between HFNC and NCPAP support was statistically significant—with PtcCO_2_ being slightly higher in HFNC support (47.9 ± 6.4 mmHg) compared to NCPAP (46.9 ± 6.0 mmHg; *P* = 0.02)—the clinical relevance of this difference may be considered minor. This suggests that while NCPAP provides more stable expiratory pressure and thus more effective CO_2_ washout, HFNC still serves as a viable option for weaning from NCPAP due to its sufficient support capabilities. Notably, most studies have focused on comparing NCPAP and HFNC support as the main respiratory therapy for premature infants after birth. Lampland et al. found no differences in the HR and arterial oxygen saturation during NCPAP and HFNC use in premature infants, but the respiratory rate was higher with the HFNC [7]. However, Taha et al. reported that HFNC use resulted in higher mortality and bronchopulmonary dysplasia rates, prolonged hospital stay, and longer time for oral feeding than NCPAP use among newborns [8]. Therefore, the American Academy of Pediatrics suggested HFNC support as an alternative respiratory therapy for infants after extubation rather than the main respiratory therapy for premature infants after birth [9, 10]. As previously mentioned, HFNC support has been suggested as an accompanying respiratory therapy for NCPAP weaning in premature infants because of the variable stability of the airway support pressure [1–3, 5, 11–15]. Consequently, we inferred that the PtcCO_2_ was higher with HFNC support than with NCPAP support, possibly due to the greater stability of the airway support pressure of NCPAP.

There were no obvious differences in cerebral StO_2_ with NCPAP or HFNC support among the 46 premature infants in our study. Sett et al. found no obvious differences in cerebral StO_2_ when performing NCPAP weaning with HFNC use in premature infants [16]. Bemdich et al. found no influence of cerebral StO_2_ and cerebral blood flow with PEEP of 3–8 cm H_2_O [17]. Combining our findings with those of the literature mentioned above, it is evident that using the HFNC for NCPAP weaning did not influence cerebral StO_2_. Therefore, the SpO_2_ and cerebral StO_2_ did not negatively influence premature infants when the HFNC was used during the NCPAP weaning process, which indicates the safety of the cyclic use of HFNC for NCPAP weaning.

Our study design involved the cyclic use of NCPAP and HFNC every 3 h for NCPAP weaning for premature infants with RDS; however, 18 (39%) premature infants required cyclic use for > 3 days to achieve complete NCPAP weaning (failure group). We inferred that lower GA, lower BW, higher NTISS, and late-onset sepsis may be associated with a higher failure rate due to the unstable condition of premature infants. However, the results showed no significant differences in clinical outcomes and complications, which may have been influenced by the small sample size (Table 3). Additionally, we were unable to measure lung function directly in these preterm infants, some of whom might inherently have delayed lung maturation. A lower cerebral StO_2_ was observed in the failure group during NCPAP weaning using the HFNC for 3 days, indicating more unstable oxygenation with HFNC support (Table 4); therefore, the infants required more number of days to achieve successful NCPAP weaning (Table 5). Sett et al. found no obvious differences in the cerebral StO_2_ when performing NCPAP weaning with 6 cm H_2_O using the HFNC at 8 L/min; however, they adjusted the fraction of inspired oxygen for the HFNC to maintain the SpO_2_ at approximately 92–95%, which could be why their results were different from ours [16]. The PMA at discontinuation of NCPAP or HFNC use was older, and the total number of days of respiratory therapy was longer in the failure group (Table 5). The length of hospitalization and PMA at discharge were higher in the failure group, and the longer respiratory therapy course might influence both groups. However, the statistical results showed no obvious difference between the success and failure groups, which might be associated with the small sample size in our study (Table 5).

This study had some limitations. The small sample size might have influenced the analysis results. In this prospective study, the HFNC devices were funded by Kaohsiung Medical University Hospital. Due to the limitations of the grant, we were only able to purchase approximately 50 devices. Additionally, this study is structured similarly to a cross-over study, rather than a comparative study, which precludes calculating the difference in treatment effects. As a result, this is a preliminary study. Previously, using HFNC was self-paid in Taiwan; however, because the health insurance of Taiwan began covering the use of the HFNC in 2022, further analyses with larger sample sizes can be considered in the future.

## Conclusions

The feasibility and safety of the weaning protocol from NCPAP using cyclic HFNC support for premature infants are acceptable. During the weaning process, there were no significant changes in HR, RR, SpO_2_, or StO_2_, although PtcCO_2_ levels were higher with HFNC support. No adverse effects related to HFNC use were observed, and there was no increase in apnea frequency. However, due to the limited number of participants, further studies with larger samples are required for more comprehensive analysis.

## Data Availability

No datasets were generated or analysed during the current study.

## Abbreviations

RDS: Respiratory distress syndrome
NICU: Neonatal intensive care unit
NCPAP: Nasal continuous positive airway pressure
HFNC: High-flow nasal cannula
PEEP: Positive end-expiratory pressure
GA: Gestational age
FiO_2_: Fraction of inspired oxygen
HR: heart rate
RR: Respiratory rate
SpO_2_: Pulse oximetry
PtcCO_2_: Transcutaneous carbon dioxide
StO_2_: Cerebral tissue oxygen saturation
BW: Body weight
PMA: Post-menstrual age
SD: Standard deviation
